# Validation of the Body Scan^®^, a new device to detect small fiber neuropathy by assessment of the sudomotor function: agreement with the Sudoscan^®^

**DOI:** 10.3389/fneur.2023.1256984

**Published:** 2023-10-31

**Authors:** Jean-Pierre Riveline, Roberto Mallone, Clarisse Tiercelin, Fetta Yaker, Laure Alexandre-Heymann, Lysa Khelifaoui, Florence Travert, Claire Fertichon, Jean-Baptiste Julla, Tiphaine Vidal-Trecan, Louis Potier, Jean-Francois Gautier, Etienne Larger, Jean-Pascal Lefaucheur

**Affiliations:** ^1^Diabetology and Endocrinology Department, Lariboisière Hospital, Paris, France; ^2^Diabetology Department, Cochin Hospital, Paris, France; ^3^Diabetology – Endocrinology and Nutrition Department, Bichat-Claude-Bernard Hospital, Paris, France; ^4^Unité de Neurophysiologie Clinique, Hôpital Henri Mondor, AP-HP, Créteil, France; ^5^EA4391 (ENT), Faculté de Santé, Université Paris Est Créteil, Créteil, France

**Keywords:** amyloidosis, autonomic testing, diabetes, diagnosis, small fiber neuropathy, sudomotor function, sympathetic nerve fibers

## Abstract

**Background:**

Sudomotor dysfunction is one of the earliest manifestations of small fiber neuropathy (SFN), reflecting the alteration of sympathetic C fiber innervation of the sweat glands. Among other techniques, such innervation can be assessed by measuring electrochemical skin conductance (ESC) in microsiemens (μS). In this study, ESC was measured at the feet to detect distal SFN. For this objective, the performance of a new device, the Body Scan^®^ (Withings, France), intended for home use, was compared with that of a reference device, the Sudoscan^®^ (Impeto Medical, France), which requires a hospital setting.

**Methods:**

In patients with diabetes with or without neuropathy or non-diabetic patients with lower-limb neuropathy, the diagnostic performance of the Body Scan^®^ measurement was assessed by calculating its sensitivity (Se) and specificity (Sp) to detect at least moderate SFN (Se70 and Sp70), defined by a value of feet ESC ≤ 70 μS and > 50 μS on the Sudoscan^®^ measure, or severe SFN (Se50 and Sp50), defined by a value of feet ESC ≤ 50 μS on the Sudoscan^®^ measure. The agreement between the two devices was assessed with the analysis of Bland–Altman plots, mean absolute error (MAE), and root mean squared error (RMSE) calculations. The repeatability of the measurements was also compared between the two devices.

**Results:**

A total of 147 patients (52% men, mean age 59 years old, 76% diabetic) were included in the analysis. The sensitivity and specificity to detect at least moderate or severe SFN were: Se70 = 0.91 ([0.83, 0.96]), Sp70 = 0.97 ([0.88, 0.99]), Se50 = 0.91 ([0.80, 0.98]), and Sp50 = 0.99 ([0.94, 1]), respectively. The bias and 95% limits of agreement were 1.5 [−5.4, 8.4]. The MAE was 2.9 and the RMSE 3.8. The intra-sample variability was 2.0 for the Body Scan^®^ and 2.3 for the Sudoscan^®^.

**Conclusion:**

The ESC measurements provided by the Body Scan^®^ were in almost perfect agreement with those provided by the reference device, the Sudoscan^®^, which validates the accuracy of the Body Scan^®^ for the detection of SFN. By enabling simple, rapid, and autonomous use by the patient at home, this new technique will facilitate screening and monitoring of SFN in daily practice.

**Clinical trial registration:**

ClinicalTrials.gov, identifier NCT05178459.

## Introduction

Small fiber neuropathy (SFN) selectively affects small-diameter peripheral nerve fibers that are thinly myelinated (A-delta) and unmyelinated (C) fibers. This type of neuropathy can be characterized by sensory symptoms (loss or decrease in temperature and nociceptive sensation, which may be accompanied by painful symptoms) and dysautonomia. This results in a negative impact on patients’ quality of life, both physically and mentally, for millions of people around the world (1–10). Since a disturbance of the autonomic nervous system can be present in SFN, autonomic testing is useful in clinical settings and includes a variety of assessment techniques (11). Although a true gold standard for the diagnosis of autonomic neuropathy is lacking, one way to detect this condition is to assess the sudomotor function in the limb extremities, as it is one of the first affected functions in SFN-associated dysautonomia (12, 13). Sudomotor testing can be based on: (i) the detection of sweating reflected by a color change of a dye applied to the skin (thermoregulatory sweat test); (ii) the evaluation of axon-reflex sweating in response to local iontophoresis of cholinergic drugs by visualizing sweat droplet imprints on a silastic (silicone) layer or by measuring sweat outflow volume with a sudorometer (Quantitative Sudomotor Axon Reflex Testing, QSART); (iii) the electrophysiological recording of sympathetic skin potentials; and (iv) the measurement of electrochemical skin conductance (ESC) by the Sudoscan^®^ device (11). The QSART technique has been used for the diagnosis of SFN (14, 15), but it requires high technical skills, is time-consuming, and its reproducibility is debated (16, 17). Thus, during the last decade, the measurement of ESC with the Sudoscan^®^ technique has been increasingly used (18), as it allows a simple and objective quantification of the distal reactivity of autonomic C fibers in the four extremities (hands and feet) by means of a rapid test (duration of 2 min). The Sudoscan^®^ technique consists of delivering a direct current (DC) at low voltage through stainless steel electrodes (anode and cathode) on which the patient applies palms and soles (19). This current causes sweating and the chloride ions released by the sweat glands on the skin surface interact with the electrodes (reverse iontophoresis). The technique is also based on chronoamperometry, with a succession of current steps (four combinations of 15 different DC incremental voltages ≤4 V) delivered at the level of the working electrode (anode). The resulting current x time function is evaluated and the device then calculates the skin conductance (ESC) expressed in microsiemens (mS). Thus, ESC values depend on the amount of chloride ions that react with the anode, and therefore reflect the reactivity of the unmyelinated C fibers innervating the sweat glands (20).

This technology has been used to assess SFN in several populations (21), primarily for the diagnosis of diabetic neuropathies in a large number of articles (22–34), but also in a wide variety of peripheral neuropathies beyond diabetes (35).

In this study, we evaluated the agreement between the Sudoscan^®^ (Impeto Medical, France) and a new device called the Body Scan^®^ (Withings, France) intended for home use, for measuring ESC at the feet. In addition to performing ESC measurement, the Body Scan^®^ is a connected body scale that records weight, full and segmental body composition, heart rate, vascular age, pulse wave velocity, and a 6-lead electrocardiogram. The glass plate is covered with indium-tin oxide electrodes. Upon stepping on the scale, weight is measured first, followed by the other measurements. The measurement of the ESC lasts less than 20 s. Results are displayed on the screen of the device and on the companion application Health Mate^®^. Neither special subject preparation nor specially trained medical personnel are required to complete the test. Only the ESC measurement function was assessed in the present study.

## Methods

### Study design

**Figure 1:**
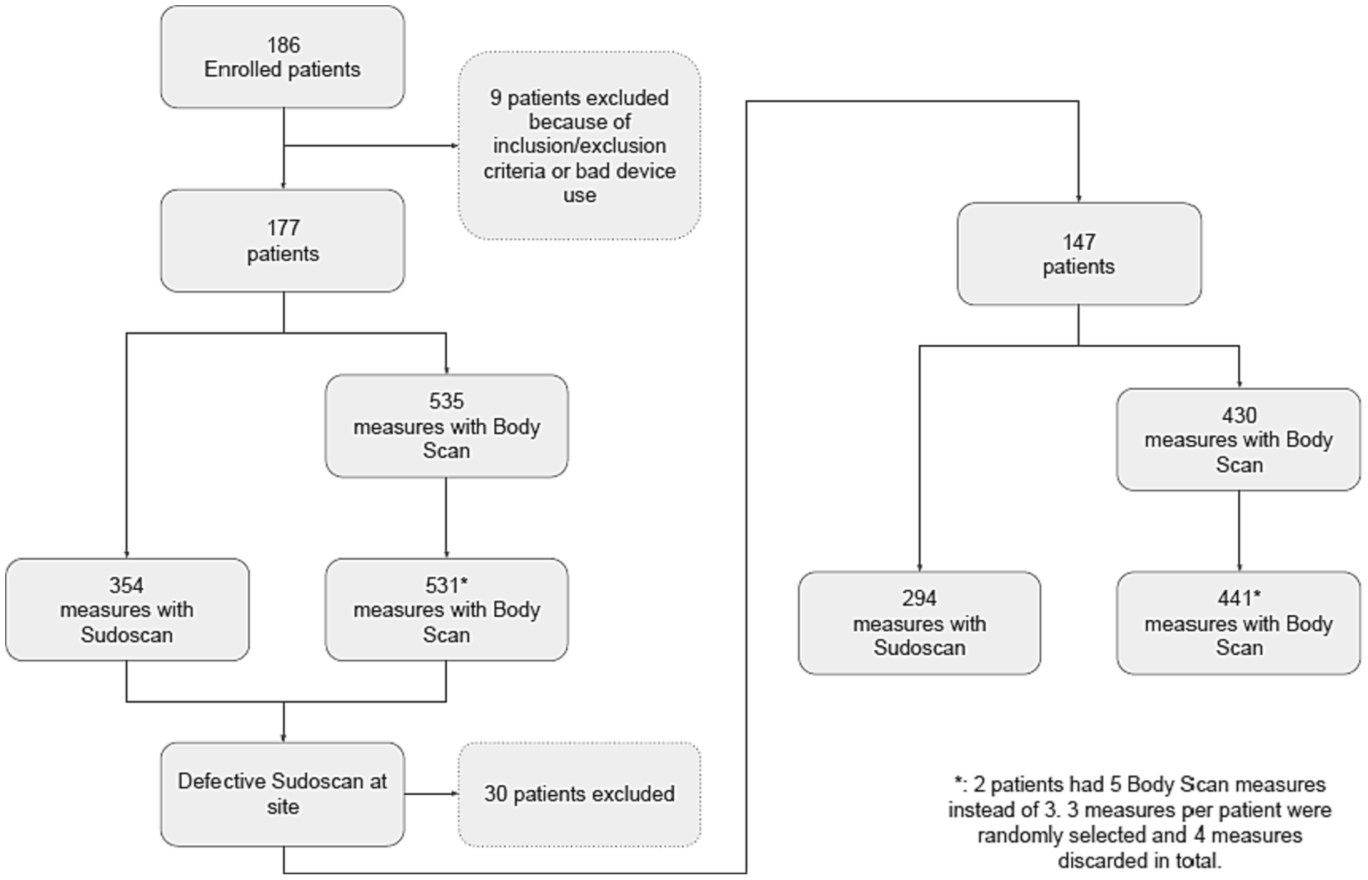
Flow chart.

This is a multicenter cross-sectional study comparing the Body Scan^®^ to the Sudoscan^®^, taken as the reference device, to measure ESC at the feet. Four sites participated in the study: the Diabetology and Endocrinology departments of three university hospitals in Paris, France (Bichat-Claude-Bernard, Cochin, and Lariboisière Hospitals), and the Clinical Neurophysiology department of Henri-Mondor University Hospital in Creteil, France (Figure 1).

Inclusion criteria were as follows: (i) being aged 18 years or older, (ii) having diabetes and/or peripheral neuropathy, (iii) being affiliated to the social security system, and (iv) consenting to participate in the study. Exclusion criteria were: (i) pregnancy, (ii) pacemaker, (iii) lower limb amputation, (iv) inability to stand still for several minutes, and (v) treatment with antidepressants.

The design of the study was to enroll approximately 75% diabetic patients, including 1/3 patients without autonomic neuropathy, 1/3 patients with moderate autonomic neuropathy, and 1/3 patients with severe autonomic neuropathy, as defined by a Sudoscan^®^ measurement performed in routine care before inclusion. According to published normative references for the Sudoscan^®^ (36), the range of ESC values were 70–100 μS for normal sudomotor function, 50–70 μS for moderate sudomotor dysfunction, and 0–50 μS for severe sudomotor dysfunction. The remaining 25% of patients were non-diabetic patients with autonomic neuropathy, moderate for half of the patients, and severe for the other half, as also defined by a prior Sudoscan^®^ measurement. A total sample of 147 patients to be analyzed was calculated, taking into account the data previously established with the Sudoscan^®^ and the four primary endpoints (see below), namely, the sensitivity (Se) and specificity (Sp) of the measurements for distinguish patients classified as having normal or impaired sudomotor function, for a type I error rate α = 0.05 and a type II error rate β = 0.05. However, the distribution between diabetic and non-diabetic patients was an arbitrary choice. Next, the study relied on consecutive sampling, a non-probability sampling technique in which each subject meeting the inclusion criteria is selected until patient recruitment is stopped when the sample size required is achieved in each of the given categories.

The study was duly approved by the National Ethics Committee (Comité de Protection des Personnes Sud-Est I), registered on ClinicalTrials.gov (NCT05178459), and conducted in accordance with good clinical practice (ISO 14155:2020, ICH guidelines) and the Declaration of Helsinki. All participants gave their written informed consent before inclusion.

### ESC measurement

The reference and test devices use the same technology to assess sudomotor function by measuring ESC based on chronoamperometry and reverse iontophoresis. However, the Sudoscan^®^ performs ESC measurements at the hands and feet while the Body Scan^®^ does so only at the feet. In our practice, patients are usually asked not to use any cream on the feet in the days preceding the examination, and in all cases the skin was thoroughly cleaned before the recordings.

In this study, each subject first performed a measurement on the Sudoscan^®^ device for 2 min, followed by three successive measurements on the Body Scan^®^ device for a total duration of approximately 1 min 30, after which a second measurement with the Sudoscan^®^ was performed, again for 2 min. Patients were instructed to stand still and not to move during the measurements.

### Medical information

Medical information was collected from a survey under medical supervision for each patient, including demographic data (gender, age, height, weight, body mass index (BMI), and ethnicity), and the presence and type of diabetes. In addition, the patients were specifically categorized in four clinical domains, according to a list of conditions checked by the investigators and detailed below:

- Cardiovascular risk factor (beyond diabetes) or previous cardiovascular event, defined as at least one of the following: hypertension, obesity, dyslipidemia, kidney failure, arrhythmia or abnormal heart rhythm, coronary artery disease (angina pectoris, heart attack), heart failure, stroke, congenital heart disease, cardiomyopathy, or valvular heart disease, among others.
- History of “surgical” intervention related to diabetes or cardiovascular disease, defined as at least one of the following: laser treatment of diabetic retinopathy or revascularization for coronary syndrome or peripheral artery syndrome, among others.
- Foot injury, defined as at least one of the following: callus, sores, blisters, skin changes, ingrown toenails, fungal skin infection, or toe deformity, among others.
- Current medications related to diabetes or cardiovascular disease, defined as at least one of the following: anti-diabetic medications, insulin replacement therapy, alpha or beta blockers, calcium channel blockers, angiotensin converting enzyme inhibitor or receptor blockers, diuretics, antiarrhythmic agents, or antiplatelet drugs or anticoagulants, among others.

### Primary endpoint

The primary objective was to determine the sensitivity (Se) and specificity (Sp) of the Body Scan^®^ device to discriminate between patients classified as having normal sudomotor function or moderate or severe sudomotor dysfunction by the Sudoscan^®^ measurement performed at the feet (normal ESC values: ⩾ 70 μS, moderate alteration: 50 μS ⩽ ESC < 70 μS, or severe alteration: ESC < 50 μS). We therefore calculated the following four outcome measures of the Body Scan^®^ measurement performance: Se70 and Sp70, for detecting the presence of at least moderate neuropathy (ESC < 70 μS with the Sudoscan^®^) and Se50 and Sp50 for detecting the presence of severe neuropathy (ESC < 50 μS with the Sudoscan^®^). In addition, the 95% confidence intervals were calculated using the Clopper–Pearson method.

### Secondary endpoints

First, the influence of the following factors: study center, ethnicity, and the presence of diabetes or foot injury on the ESC measurements made with the Body Scan^®^ were assessed using one-way analysis of variance (ANOVAs).

Second, the agreement between the ESC measurements made with the Body Scan^®^ and the Sudoscan^®^ devices was assessed by the Bland–Altman method (37, 38) with the calculation of the bias (paired Body Scan^®^ minus Sudoscan^®^ measures) and the 95% limits of agreement. The mean absolute error (MAE) and root mean square error (RMSE) were also calculated as follows:


$$MAE=\frac{1}{n}\overset{n}{\underset{i=1}{\sum }}\left|ESC-ESC_{ref}\right|$$

$$\mathrm{RMSE}=\frac{1}{n}\overset{n}{\underset{i=1}{\sum }}{\left({\left(ESC-ESC_{ref}\right)}^{2}\right)}^{\frac{1}{2}}$$

where ESC was the value measured with the Body Scan^®^, ESC_ref_ was the value measured with the Sudoscan^®^, and *n* was the total number of measurements.

Third, the repeatability of the ESC measurements made with the Body Scan^®^ or the Sudoscan^®^ device was assessed using an ANOVA with the “measure” and the “subject” as fixed factors. For this analysis, the three measurements made with the Body Scan^®^ and the two measurements made with the Sudoscan^®^ were considered. The repeatability was expressed as the intra-sample standard deviation (SD) or variability, calculated with the variance method from the within-subjects sum of squares.

### Statistical methods

Descriptive data are presented as mean (SD) or number (percentage). For all analyses (except repeatability assessment), the average of the three measurements made in both feet with the Body Scan^®^ device and that of the two measurements made in both feet with the Sudoscan^®^ device were taken into account for each patient. In addition, beyond the whole series, subgroup analyses were also performed to assess the influence of the following factors: study center, ethnicity, and the presence of diabetes or foot injury. Outliers were defined as values above [Q3 + 1.5xIQR] or below [Q1 – 1.5xIQR], with Q1 and Q3 being the first and third quartile, respectively, and IQR being the interquartile range (Q3 – Q1). Extreme outliers were defined as values above [Q3 + 3xIQR] or below [Q1 − 3xIQR]. Normality assumptions before ANOVAs were verified using the Shapiro–Wilk test. Statistical analyses were performed with R software packages.

## Results

### Study population

A total of 186 patients were included. Nine patients were excluded due to protocol deviations related to inclusion/exclusion criteria and device misuse. Thirty more patients were excluded in one study center due to a technical defect in the Sudoscan^®^ device, detected by an excessive variability in individual measurements. This turned out to be related to a failure of the calibration system of the Sudoscan^®^ device used and this led to its replacement for the remainder of the study in this center. Hence, 147 patients were included in the analysis, which was the expected sample size. The flow diagram of the study is presented in Figure 1. No adverse effects were observed with either device.

Demographic and clinical characteristics of the study population are summarized in Table 1. Overall, the male-to-female sex ratio was 1.1, mean age was 58.9 (13.6) years, and mean BMI was 27.2 (5.2). The most represented ethnic groups were Caucasian (39%), North African (30%) and Sub-Saharan African (22%). There were 76% of diabetic patients, mostly type 2 (78%) with an average HbA1C of 8.1 (1.9) %. In the study center not involved in the recruitment of diabetic patients (Henri Mondor center), SFN was related to amyloidosis (61%), associated to familial transthyretin mutation or abnormal immunoglobulin light chain, primary Sjögren’s syndrome (8%), or a not fully characterized inflammatory or dysimmune cause (31%). A cardiovascular risk factor (beyond diabetes) was present in 83% of patients, with at least one prior cardiovascular event in 23% of patients. A history of “surgical” intervention related to diabetes or cardiovascular disease was present in 30% of patients and foot injury in 38% of patients. Medications related to diabetes or cardiovascular disease were present in 96% of patients. Results are presented according to the study centers in Table 1 and to the presence of diabetes or foot injury in Table 2.

**Table 1:** Demographic and clinical characteristics in the whole series and according to the study center.

		Total	Bichat	Cochin	Lariboisière	Mondor
Number of patients		147	49	21	41	36
Gender	*Man*	77 (52%)	31 (63%)	8 (38%)	24 (59%)	14 (39%)
	*Woman*	70 (48%)	18 (37%)	13 (62%)	17 (41%)	22 (61%)
Age (years)		58.9 (13.6)	61.2 (11.3)	59.7 (16.0)	58.0 (15.0)	56.4 (13.4)
Height (cm)		168.3 (9.2)	167.9 (9.4)	167.0 (8.0)	170.2 (9.3)	167.3 (9.7)
Weight (kg)		76.9 (15.5)	81.1 (14.6)	78.1 (15.4)	75.6 (16.9)	71.9 (14.1)
Body mass index		27.2 (5.2)	28.8 (4.9)	28.0 (5.5)	26.0 (5.3)	25.7 (4.7)
Ethnicity	*Caucasian*	58 (39%)	11 (22%)	10 (48%)	12 (29%)	25 (69%)
	*North African*	44 (30%)	23 (47%)	5 (24%)	12 (29%)	4 (11%)
	*Sub-Saharan African*	32 (22%)	11 (22%)	4 (19%)	12 (29%)	5 (14%)
	*Asian*	8 (5.4%)	3 (6.1%)	0 (0%)	5 (12%)	0 (0%)
	*Other*	5 (3.4%)	1 (2.0%)	2 (9.5%)	0 (0%)	2 (5.6%)
Diabetes	*Total number*	111 (76%)	49 (100%)	21 (100%)	41 (100%)	0 (0%)
	*Type 1*	24 (22%)	8 (16%)	8 (38%)	8 (20%)	0 (0%)
	*Type 2*	87 (78%)	41 (84%)	13 (62%)	33 (80%)	0 (0%)
	*HbA1C (%)*	8.1 (1.9)	7.8 (1.7)	9.5 (2.1)	7.6 (1.7)	NA (NA)
Cardiovascular risk	*No*	25 (17%)	2 (4.1%)	3 (14%)	5 (12%)	15 (42%)
	*At least one*	122 (83%)	47 (96%)	18 (86%)	36 (88%)	21 (58%)
Cardiovascular event	*No*	113 (77%)	46 (94%)	16 (76%)	34 (83%)	17 (47%)
	*At least one*	34 (23%)	3 (6.1%)	5 (24%)	7 (17%)	19 (53%)
Surgical intervention	*No*	103 (70%)	30 (61%)	11 (52%)	26 (63%)	36 (100%)
	*At least one*	44 (30%)	19 (39%)	10 (48%)	15 (37%)	0 (0%)
Foot injury	*No*	91 (62%)	9 (18%)	16 (76%)	31 (76%)	35 (97%)
	*At least one*	56 (38%)	40 (82%)	5 (24%)	10 (24%)	1 (2.8%)
Medications	*No*	6 (4.1%)	0 (0%)	0 (0%)	0 (0%)	6 (17%)
	*At least one*	141 (96%)	49 (100%)	21 (100%)	41 (100%)	30 (83%)

Data are presented as mean (standard deviation) or number (percentage).

**Table 2:** Demographic and clinical characteristics in the whole series and according to the presence or absence of diabetes or foot injury.

		Diabetes	No diabetes	Foot injury	No foot injury
Number of patients		111	36	56	91
Study center	*Bichat*	49 (44%)	0 (0%)	40 (71%)	9 (9.9%)
	*Cochin*	21 (19%)	0 (0%)	5 (8.9%)	16 (18%)
	*Lariboisière*	41 (37%)	0 (0%)	10 (18%)	31 (34%)
	*Mondor*	0 (0%)	36 (100%)	1 (1.8%)	35 (38%)
Gender	*Man*	63 (57%)	14 (39%)	30 (54%)	47 (52%)
	*Woman*	48 (43%)	22 (61%)	26 (46%)	44 (48%)
Age (years)		59.7 (13.7)	56.4 (13.4)	62.3 (11.9)	56.8 (14.2)
Height (cm)		168.6 (9.1)	167.3 (9.7)	167.1 (9.3)	169.0 (9.2)
Weight (kg)		78.5 (15.7)	71.9 (14.1)	79.7 (15.5)	75.2 (15.4)
Body mass index		27.6 (5.3)	25.7 (4.7)	28.6 (5.4)	26.3 (4.9)
Ethnicity	*Caucasian*	33 (30%)	25 (69%)	12 (21%)	46 (51%)
	*North African*	40 (36%)	4 (11%)	27 (48%)	17 (19%)
	*Sub-Saharan African*	27 (24%)	5 (14%)	12 (21%)	20 (22%)
	*Asian*	8 (7.2%)	0 (0%)	4 (7.1%)	4 (4.4%)
	*Other*	3 (2.7%)	2 (5.6%)	1 (1.8%)	4 (4.4%)
Diabetes	*Total number*	111 (100%)	0 (0%)	55 (98%)	56 (62%)
	*Type 1*	24 (22%)	0 (0%)	7 (13%)	17 (30%)
	*Type 2*	87 (78%)	0 (0%)	48 (87%)	39 (70%)
	*HbA1C (%)*	8.1 (1.9)	NA (NA)	8.1 (2.0)	8.0 (1.8)
Cardiovascular risk	*No*	10 (9.0%)	15 (42%)	4 (7.1%)	21 (23%)
	*At least one*	101 (91%)	21 (58%)	52 (93%)	70 (77%)
Cardiovascular event	*No*	96 (86%)	17 (47%)	50 (89%)	63 (69%)
	*At least one*	15 (14%)	19 (53%)	6 (11%)	28 (31%)
Surgical intervention	*No*	67 (60%)	36 (100%)	29 (52%)	74 (81%)
	*At least one*	44 (40%)	0 (0%)	27 (48%)	17 (19%)
Foot injury	*No*	56 (50%)	35 (97%)	0 (0%)	91 (100%)
	*At least one*	55 (50%)	1 (2.8%)	56 (100%)	0 (0%)
Medications	*No*	0 (0%)	6 (17%)	0 (0%)	6 (6.6%)
	*At least one*	111 (100%)	30 (83%)	56 (100%)	85 (93%)

Data are presented as mean (standard deviation) or number (percentage).

### Sensitivity and specificity of the ESC measurements made with the Body Scan^®^

In the whole series of patients, the sensitivity and specificity values ([95% confidence intervals]) for detecting the presence of sudomotor dysfunction, at least moderate, with the Body Scan^®^ device were Se70 = 0.91 ([0.83, 0.96]) and Sp70 = 0.97 ([0.88, 0.99]) with corresponding likelihood ratios (LR) of LR70+ = 26.4 and LR70− = 0.09, respectively. For detecting severe sudomotor dysfunction, the values were Se50 = 0.91 ([0.80, 0.98]) and Sp50 = 0.99 ([0.95, 1.0]) with LR50+ = 91.5 and LR50− = 0.09, respectively. Table 3 presents the Se70, Sp70, Se50, and Sp50 in the whole series of patients and according to the study center, ethnicity, and the presence of diabetes or foot injury.

**Table 3:** Sensitivity (Se) and specificity (Sp) of the Body Scan^®^ device for detecting the presence of at least moderate or severe sudomotor dysfunction according to the value of electrochemical skin conductance (< 70 or 50 μS, respectively).

		Se_70_	Sp_70_	Se_50_	Sp_50_
Whole series	*147 patients*	0.910	0.966	0.915	0.990
Study center	*Bichat*	0.968	1	0.867	1
	*Cochin*	1	0.846	1	1
	*Lariboisière*	0.880	1	0.933	1
	*Mondor*	0.840	1	0.917	0.958
Ethnicity	*Caucasian*	0.929	0.967	0.875	1
	*North African*	0.826	0.936	1	1
	*Sub-Saharan African*	0.960	1	0.944	0.929
	*Asian*	1	1	0.75	1
	*Other*	0.75	1	1	1
Diabetes	*Present*	0.938	0.957	0.914	1
	*Absent*	0.840	1	0.917	0.958
Foot injury	*Present*	0.974	0.941	0.905	1
	*Absent*	0.860	0.976	0.923	0.984

### Influence of study center, ethnicity, and the presence of diabetes or foot injury on the ESC measurements with the Body Scan^®^

Table 4 presents the influence of the following factors: study center, ethnicity, and the presence of diabetes or foot injury on the ESC measurements made with the Body Scan^®^ assessed using one-way ANOVAs. ESC values did not differ between study centers or by the presence or absence of diabetes. In contrast, ESC values differed by ethnic group and the presence or absence of foot injury. To confirm that ethnicity and foot injury had independent effects on ESC measures, we performed a two-way ANOVA, which confirmed that both factors were significant. On the one hand, ESC values were lower in Asian and Sub-Saharan patients than in the other ethnic groups, and on the other hand, ESC values were lower in the presence of a foot injury than in the absence of a foot injury.

**Table 4:** Influence of study center, ethnicity, and the presence or absence of diabetes or foot injury on the electrochemical skin conductance (ESC) values measured with the Body Scan^®^ device.

		ESC measure	ANOVA Df	Mean square	*F* value	*p* value
Study center	*Bichat*	61.2 (16.7)	3	503	1.28	0.41
	*Cochin*	65.1 (19.8)				
	*Lariboisière*	61.9 (20.6)				
	*Mondor*	59.1 (22.4)				
Ethnicity	*Caucasian*	65.9 (19.4)	4	5685.41	17.28	<1.0e-06
	*North African*	65.8 (18.5)				
	*Sub-Saharan African*	48.6 (18.4)				
	*Asian*	56.0 (14.9)				
	*Other*	63.3 (12.3)				
Diabetes	*Present*	62.2 (18.8)	1	26	0.06	0.15
	*Absent*	59.1 (22.4)				
Foot injury	*Present*	57.1 (19.1)	1	5716.20	17.37	1.0e-04
	*Absent*	64.1 (19.7)				

ESC values are presented as mean (standard deviation) in μS.

### Agreement between the ESC measurements made with the Body Scan^®^ and the Sudoscan^®^

**Figure 2:**
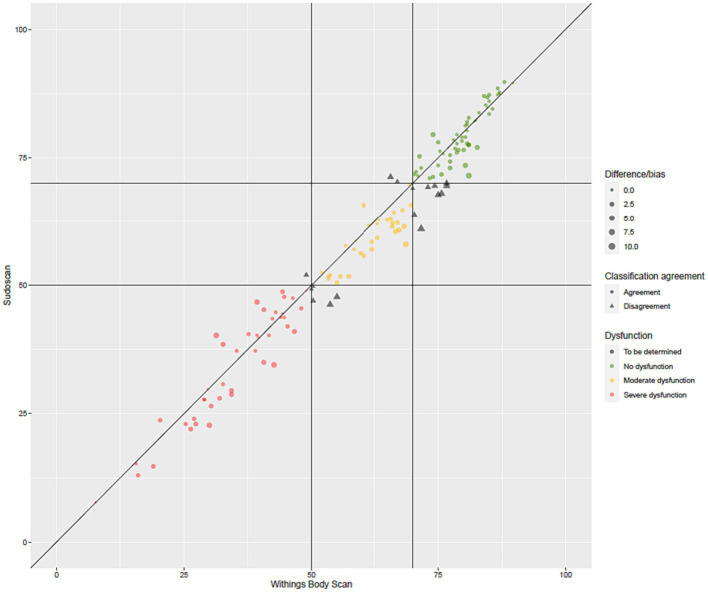
Agreement plot of the electrochemical skin conductance values (in μS) measured with the Body Scan^®^ device versus the Sudoscan^®^ device.

**Figure 3:**
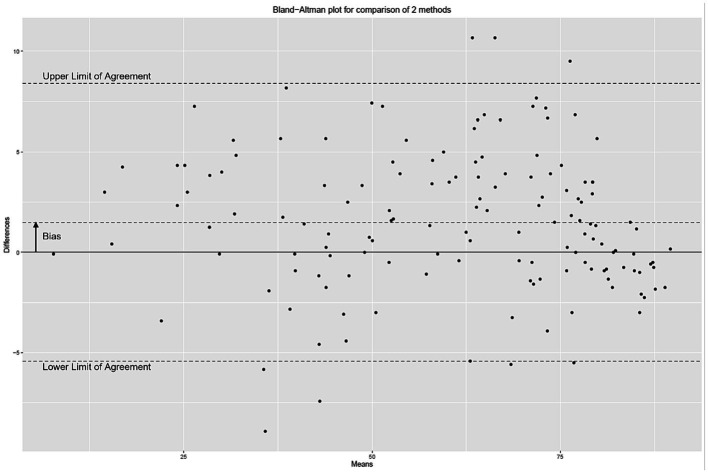
Bland–Altman plot of the electrochemical skin conductance values (in μS) measured with the Body Scan^®^ device minus the Sudoscan^®^ device.

Figure 2 presents the distribution of ESC values measured with the Body Scan^®^ device versus the Sudoscan^®^ device, showing the excellent agreement between the two measurements. Only one patient (LA-034) was found to be an outlier (*cf.* definition in methods), while no extreme outliers were observed. The Bland–Altman plot is shown on Figure 3. In the whole series of patients, the bias (paired Body Scan^®^ minus Sudoscan^®^ measures) was 1.5 μS with 95% limits of agreement of [−5.4, 8.4], the MAE was 2.9, and the RMSE was 3.8. Table 5 presents the agreement data in the whole series of patients and according to the study center, ethnicity, and the presence of diabetes or foot injury.

**Table 5:** Agreement between the electrochemical skin conductance values measured with the Body Scan^®^ and the Sudoscan^®^ devices.

		Bias	LOA	MAE	RMSE
Whole series	*147 patients*	1.5	[−5.4, 8.4]	2.9	3.8
Study center	*Bichat*	1.5	[−4.3, 7.1]	2.5	3.2
	*Cochin*	0.04	[−6.9, 7.0]	2.7	3.5
	*Lariboisière*	0.8	[−5.6, 7.2]	2.5	3.4
	*Mondor*	3.2	[−4.3, 10.7]	4.2	5.0
Ethnicity	*Caucasian*	2.1	[−5.2, 9.4]	3.5	4.3
	*North African*	1.4	[−4.2, 7.0]	2.4	3.2
	*Sub-Saharan African*	0.4	[−6.6, 7.6]	2.7	3.6
	*Asian*	1.2	[−3.9, 6.2]	2.3	2.8
	*Other*	3.8	[−4.2, 11.9]	4.2	5.6
Diabetes	*Present*	0.9	[−5.2, 7.3]	2.5	3.3
	*Absent*	3.2	[−4.3, 10.7]	4.2	5.0
Foot injury	*Present*	0.9	[−5.3, 7.2]	2.5	3.3
	*Absent*	1.8	[−5.3, 9.0]	3.2	4.1

Bias, paired Body Scan^®^ minus Sudoscan^®^ measures; LOA, 95% limits of agreement; MAE, mean absolute error; RMSE, root mean square error.

### Repeatability of the ESC measurements made with the Body Scan^®^ and the Sudoscan^®^

**Figure 4:**
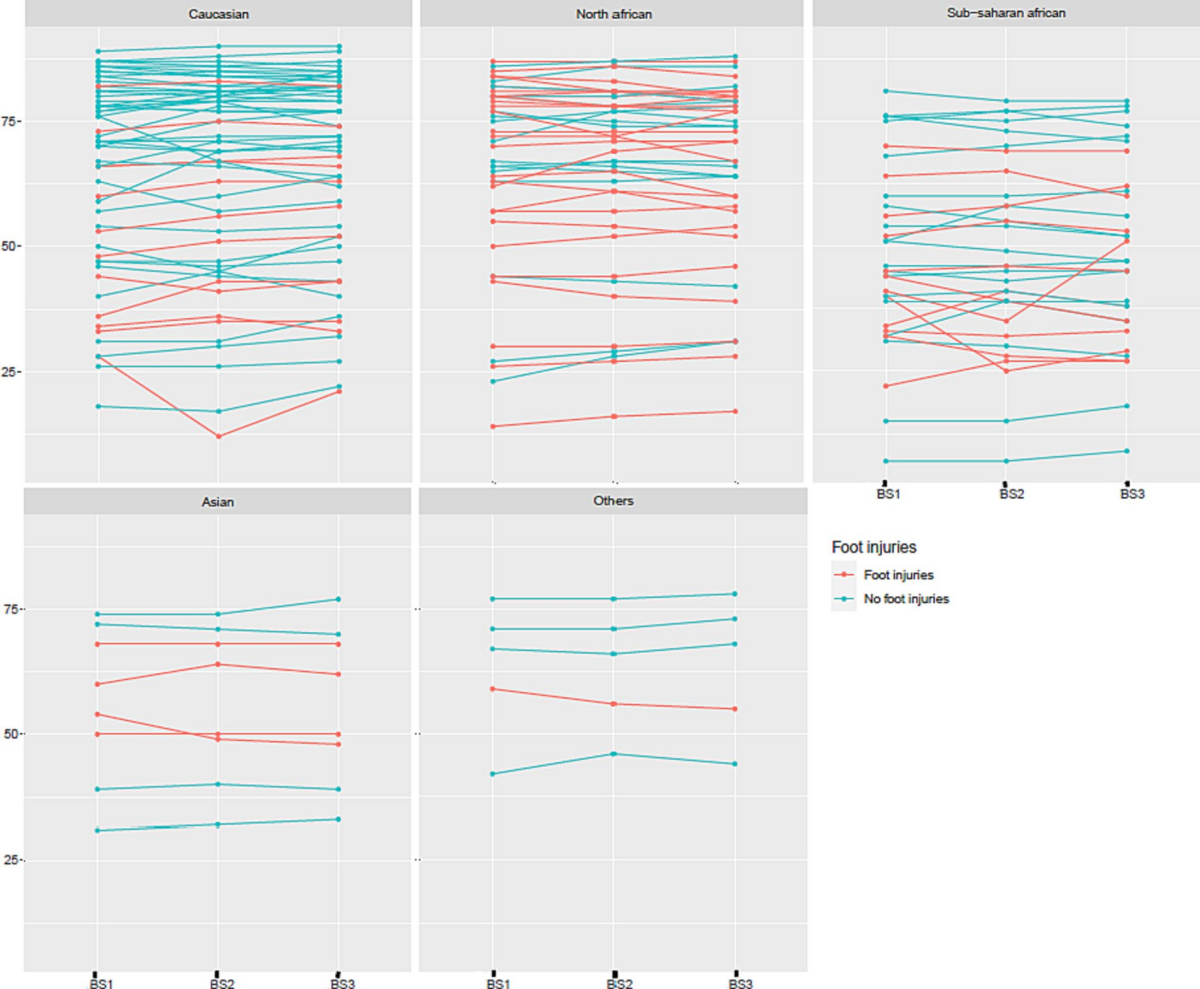
Repeatability of three measures of electrochemical skin conductance (in μS) with the Body Scan^®^ device (BS1, BS2, and BS3) according to ethnic group and the presence or absence of foot injuries.

For both the Body Scan^®^ and the Sudoscan^®^ devices, ANOVA showed no significant effect of the measure (“repetition” factor), but an effect of the subject. For the Body Scan^®^ device, detailed ANOVA results were: Df = 2, Sum of Squares = 8.91, Mean Square = 4.45, *F* value = 0.84, and *p* value = 0.43 for the “measure” factor and Df = 146, Sum of Squares = 1.69e+05, Mean Square = 1165.60, *F* value = 220.48, and *p* value <0.0001 for the “subject” factor. The repeatability of ESC measures, expressed as the intra-sample SD (variability), was 2.0 with the Body Scan^®^ device and 2.3 with the Sudoscan^®^ device. The repeatability data obtained with the Body Scan^®^ device are presented in Figure 4 according to the various ethnic groups.

## Discussion

This is the first study evaluating the performance of a new device, the Body Scan^®^, to assess sudomotor function by measuring ESC at the feet. The values obtained with the Body Scan^®^ were compared in the same patients with those obtained in the same session with the reference device, the Sudoscan^®^ which uses the same measurement principles.

The primary endpoint was the evaluation of the sensitivity and specificity of the Body Scan^®^ to reveal moderate or severe autonomic neuropathy defined by the alteration of ESC values measured at the feet with the Sudoscan^®^ device, with thresholds set at 50 and 70 μS, respectively. Using these thresholds, the sensitivities and specificities of the Body Scan^®^ device were > 0.9 in the whole series of patients, revealing the almost perfect agreement between the two devices (convergent validity). These thresholds were used as defined in the Caucasian population in a broad set of normative values (36). In this previous study, African American, Indian, and Chinese populations had lower normal ESC values (36). In the present study, we confirmed that the ESC values in the Sub-Saharan African and Asian patient groups were lower than those measured in the Caucasian group, which on the other hand were similar to the North African group, as well as to patients from “other” (including mixed) ethnicities. This inter-ethnic difference was not taken into account in the definition of the primary endpoints of sensitivity and specificity, neither for the measurements performed with the Body Scan^®^ nor for those performed with the Sudoscan^®^. Also, even if this “ethnic” factor was indeed evaluated and highlighted in the results, this cannot have influenced the existence of an agreement between the two techniques. This is the same for the presence of foot injury, which can induce a decrease in ESC measures. This significant factor of variation, never highlighted before, must be underlined for the interpretation of the results in clinical practice in the future.

It should also be noted that this study does not suffer from any bias related to the use of drugs with muscarinic antagonist activity, which could possibly impact ESC measures based on the cholinergic innervation of the sweat glands. Indeed, no patient included in this study actually received anticholinergic medications, such as those used in the cardiorespiratory field or for the treatment of overactive bladder or Parkinson’s or Alzheimer’s disease, or other medications, such as antipsychotics or tricyclics (exclusion criteria), which may have incidental muscarinic antagonist activity.

The ESC measurements provided by the Body Scan^®^ were in almost perfect agreement with those provided by the reference device, the Sudoscan^®^, which validates the reliability of the Body Scan^®^ for the detection of SFN with autonomic impairment of C fibers. However, it must be emphasized that this study was based on the definition of the existence of peripheral neuropathy and its severity based solely on the demonstration of sudomotor dysfunction in the feet. This definition and classification did not take into account the evaluation of other neuropathic modalities, for example, sensory or motor. No specific clinical score for peripheral neuropathy was performed. It is certain that a more in-depth validation of Body Scan^®^ measurements in the diagnosis and monitoring of peripheral neuropathies should be considered in future studies in the context of neuropathies better defined on a multimodal level, by both clinical examination and laboratory investigations.

The mean bias between the Body Scan^®^ minus Sudoscan^®^ measures was very low (1.5 μS), smaller than variations that can be measured between several measurements performed on the same day, estimated between 5 and 7% (39). This small positive bias shows that the Body Scan^®^ may slightly overestimate the ESC compared with the Sudoscan^®^ and explains that specificity values were higher than sensitivity values.

Both devices were also similar regarding repeatability (intra-sample SD variability = 2.0 for the Body Scan^®^ versus 2.3 for the Sudoscan^®^), consistent with the literature (intra-patient SD variability = 2.1 for the Sudoscan^®^ in Bordier et al. (40)). Unfortunately, we did not assess the reproducibility of the technique, which would have consisted of measuring the ESC values in each patient on at least two different Body Scan^®^ devices.

Thus, the measurement of ESC values at the level of the feet with the Body Scan^®^ device appears as reliable and repeatable as that performed with the Sudoscan^®^. It is important to mention that ESC is not the measure of an absolute value of conductance, but a score, ranging between 0 and 100, linked to a non-linear scale chosen to improve the diagnostic sensitivity in the range of moderate sudomotor dysfunction. The diagnostic performance of ESC measurement with the Body Scan^®^ device was assessed in the present study in patients with SFN of various degrees of severity (mild to severe) and of various origins, diabetic or non-diabetic, including amyloid neuropathy, which is a major indication for the Sudoscan^®^ technique (41–43). It is therefore important to specify that the Body Scan^®^ technique is effective, regardless of the underlying pathophysiological cause of the neuropathy.

The measurement of ESC values with the Sudoscan^®^ technique has proven to be very sensitive and specific for the diagnosis of SFN (22, 23, 28, 44, 45), even compared with other techniques with high diagnostic accuracy for sudomotor dysfunction, such as QSART (23, 26, 34) or sympathetic skin response recording (46, 47).

However, there are several major differences between the Body Scan^®^ and the Sudoscan^®^. One is to the advantage of the Sudoscan^®^, which can also measure ESC values at the hands, whereas the Body Scan^®^ measurements are limited to the feet. Two are to the advantage of the Body Scan^®^, which are the very brief duration of the measurement (30 s versus 2 min) and above all the fact that the ESC values can be measured at home by the patients themselves. This is a very significant improvement in terms of the management of patients with a chronic disease that may be associated with length-dependent SFN, such as diabetes or amyloidosis. Indeed, patients will be able to regularly follow the evolution of their neuropathy without having to come to the hospital or clinic for measurements. In addition, the Body Scan^®^ can also provide several other physiological markers, which may help raise awareness among patients of the adverse metabolic conditions that are involved in the progression of SFN.

Thus, in conclusion, more than 10 years after the first reports showing the value of evaluating sudomotor function using the Sudoscan^®^ for the diagnosis of diabetic neuropathy (48, 49) and the validation of this technique in the armamentarium of autonomic testing (50), the Body Scan^®^ appears to be a significant improvement in this field of investigation. We found the Body Scan^®^ to be as accurate and precise as the Sudoscan^®^ in the automated measurement of sudomotor function. By allowing simple, rapid, and independent use of the testing device by the patient at home, this new technique will facilitate the performance of a longitudinal assessment of the autonomic nervous system in clinical practice. However, the value of this device for early detection of peripheral neuropathy and monitoring its progression must be further validated in future studies with a larger sample, different populations presenting or not with a neuropathic condition of various origins and assessed by other modalities, and according to long-term follow-up.

## Data availability statement

The raw data supporting the conclusions of this article will be made available by the authors, without undue reservation.

## Ethics statement

The studies involving humans were approved by Comité de Protection des Personnes Sud-Est I. The studies were conducted in accordance with the local legislation and institutional requirements. The participants provided their written informed consent to participate in this study.

## Author contributions

J-PR: Conceptualization, Investigation, Methodology, Writing – review & editing. RM: Investigation, Writing – review & editing. CT: Investigation, Writing – review & editing. FY: Investigation, Writing – review & editing. LA-H: Investigation, Writing – review & editing. LK: Investigation, Writing – review & editing. FT: Investigation, Writing – review & editing. CF: Investigation, Writing – review & editing. J-BJ: Investigation, Writing – review & editing. TV-T: Investigation, Writing – review & editing. LP: Conceptualization, Writing – review & editing. J-FG: Conceptualization, Writing – review & editing. EL: Conceptualization, Writing – review & editing. J-PL: Conceptualization, Investigation, Methodology, Supervision, Writing – original draft, Writing – review & editing.
